# Classification of 5-HT_1A_ Receptor Ligands on the Basis of Their Binding Affinities by Using PSO-Adaboost-SVM

**DOI:** 10.3390/ijms10083316

**Published:** 2009-07-29

**Authors:** Zhengjun Cheng, Yuntao Zhang, Changhong Zhou, Wenjun Zhang, Shibo Gao

**Affiliations:** Institute of Applied Chemistry, China West Normal University, Nanchong 637002, Sichuan, China; E-Mails: ncczj@yahoo.com.cn (Z.-J.C.); nczyt@yahoo.com.cn (Y.-T.Z.)

**Keywords:** classification, 5-HT_1A_ selective ligands, topological descriptor, particle swarm optimization, Adaboost-SVM

## Abstract

In the present work, the support vector machine (SVM) and Adaboost-SVM have been used to develop a classification model as a potential screening mechanism for a novel series of 5-HT_1A_ selective ligands. Each compound is represented by calculated structural descriptors that encode topological features. The particle swarm optimization (PSO) and the stepwise multiple linear regression (Stepwise-MLR) methods have been used to search descriptor space and select the descriptors which are responsible for the inhibitory activity of these compounds. The model containing seven descriptors found by Adaboost-SVM, has showed better predictive capability than the other models. The total accuracy in prediction for the training and test set is 100.0% and 95.0% for PSO-Adaboost-SVM, 99.1% and 92.5% for PSO-SVM, 99.1% and 82.5% for Stepwise-MLR-Adaboost-SVM, 99.1% and 77.5% for Stepwise-MLR-SVM, respectively. The results indicate that Adaboost-SVM can be used as a useful modeling tool for QSAR studies.

## 1. Introduction

Selective serotonin (5-HT) is an important neurotransmitter that mediates various physiological and pathological processes in the peripheral and central nervous system by interaction with several different receptors [1]. To date, 14 serotonin receptor subtypes (5-HTRs) with seven subfamilies (5-HT_1–7_) have been identified on the basis of molecular cloning, amino acid sequence, pharmacology, and signal transduction [2]. Among the 5-HTRs, the 5-HT_1A_ subtype is a high affinity serotonin receptor expressed on both the neurons and nonneuronal cells throughout the brain and the spinal cord [3]. In both rodents and humans, the 5-HT_1A_ receptor [4] is highly concentrated in cortical and limbic brain areas associated with memory functions. And it is located both pre- and postsynaptically, drugs acting at the 5-HT_1A_ receptor can, depending on the dosages, inhibit or enhance 5-HT_1A_ receptor function, resulting in varying modulatory effects on key neurotransmitters (glutamate, GABA, and acetylcholine (ACh)) involved in cognition [4]. For example, the 5-HT_1A_ receptor partial agonist S15535 facilitated memory function in a number of behavioral models [5], because the actions of S15535 involved stimulation of the somatodendritic 5-HT_1A_ autoreceptors and blockade of postsynaptic 5-HT_1A_ receptors in the frontal cortex and hippocampus. Furthermore, 5-HT_1A_ receptor antagonists such as WAY-100635 can, in a dose-dependent manner, improve memory function, because it increases the basal (non-stimulated) ACh release in the cortical and hippocampal areas of the rat brain [5]. Recently, Bert *et al.* [6] reported increasing the number of 5-HT_1A_-receptors in cortex and hippocampus does not induce mnemonic deficits in mice. The dosages of drugs can affect its performance, such as, at higher doses, the full 5-HT_1A_-receptor agonist 8-OH-DPAT was found to impair learning, most likely due to activation of postsynaptic sites [6]. However, low doses of 8-OH-DPAT can improve learning and memory, because it can reduce 5-HT release in the projection areas of the raphe nuclei [4] and hippocampal 5-HT release in anaesthetized rats [7]. Moreover, different 5-HT_1A_ receptor antagonists in the rat have reported facilitation [8], impairment [9] or no effects [10] on cognitive performance in various tasks. These contradictory findings may be explained by different behavioral procedures, route of administration, and differential affinities for pre- and postsynaptic 5-HT_1A_ receptors, as well as lack of receptor specificity of the 5-HT_1A_ receptor antagonists used in the different studies. Notable, enhanced brain 5-HT activity improves memory in animals and humans whereas decreasing brain 5-HT levels by acute 5-HT depletion has been shown to impair it [11]. Hence, strategies discriminating the above sources of serotonergic tone will also contribute to the *in vivo* assessment of inverse agonist [12], agonist or antagonist effects, with 5-HT_1A_ receptors being a good candidate considering their tone as well as pre- and postsynaptic localization. Several potent 5-HT_1A_ ligands belong to different chemical classes such as arylpiperazine compounds [13], 4-halo-6-[2-(4-arylpiperazin-1-yl)ethyl]-1H-benzimidazoles [14], piperazine-pyridazinone derivatives [15], [[(arylpiperazinyl) alkyl]thio]thieno[2,3-d]pyrimidinone derivatives [16], 3-[(4-aryl) piperazin-1-yl]-1-arylpropane derivatives [17], 4-[2-(3-methoxyphenyl) ethyl]-1-(2-methoxyphenyl) piperazine [18], and arylpiperazinylalkylthiobenzimidazole, benzothiazole, or benzoxazole derivatives [1].

In previous studies, many groups were more interested in the synthesis of novel compounds as selective 5-HT_1A_ Serotonin Receptor Ligands, but time and cost considerations do not make it feasible to carry out binding bioassays on every molecule. Alternatively, an untested molecule might be evaluated using the information from already obtained bioassays and the ability to build quantitative structure activity relationships (QSAR) modeling. QSAR modeling seeks to discover and use mathematical relationships between chemical structure and biological activity. The approach does not depend on experimental data of novel compounds as selective 5-HT_1A_ Ligands, and need the molecular descriptors of the compounds, which can be calculated from the molecular structure alone. Once the structure of a compound is known, any molecular descriptor can be calculated no matter whether the compound is synthesized or not. When a model is established, we can use it to predict the properties of compounds and see which structural factors influence those properties. In order to establish the QSAR model, we should use appropriate molecular descriptors and select suitable modeling methods, including linear methods or nonlinear methods such as LDA (Linear Discriminant Analysis) [13], Spectral-SAR Algorithm [19,20], MLR (Multiple Linear Regression), PCA (Principal Component Analysis), HCA (Hierarchical Cluster Analysis), KNN (K-nearest Neighbor), PLS (Partial Least Squares), SIMCA (Soft Independent Modeling of Class Analogy), different types of artificial neural networks (ANN), SVM (Support Vector Machines), genetic algorithms (GA), HM (The Heuristic Method), and Stepwise -MLR (Stepwise Multiple Linear Regression). They can be selected in the development of a mathematical relationship between the structural descriptors of compounds and the corresponding activity of compounds.

In the past several years, many authors have studied 5-HT_1A_ receptor ligands using different QSAR models. Chilmonczyk *et al.* proposed a 3D QSAR model for various classes of 5-HT receptor ligands by applying molecular electrostatic potential [21]. Borosy *et al.* employed 3D QSAR analysis of a novel set of pyridazinothiazepines and pyridazinooxazepines with moderate-to-high affinity to 5-HT_1A_-receptors, whose model identified by DISCO (DIStance Comparison) served as a suitable mode of superposition for subsequent comparative molecular field analysis [22]. Later, Menziani *et al.* designed a theoretical QSAR model based on theoretical descriptors, ad hoc defined size and shape descriptors. And theoretical descriptors derived by means of the program CODESSA and ad hoc defined size and shape descriptors have been employed for deciphering, on a quantitative ground, the molecular features responsible for affinity and selectivity in a series of potent N_4_-substituted arylpiperazines antagonists acting at postsynaptic 5-HT_1A_ displaying a wide range of selectivity towards the α1-adrenoceptors [23]. Guccione *et al.* reported 5-HT_1A_ and α1-adrenergic receptor (α1-AR) receptor binding properties of a series of 23 thienopyrimidinones using HASL 3D-QSAR models. And the multiconformer 3D-QSAR model was demonstrated to yield robust cross-validated models for the 23 thienopyrimidinones, which were more predictive than models based on single conformers. Furthermore, the model can avoid the alignment problems typical to 3D-QSAR analyses, and it can represent advancement over other alignment-based methods in avoiding artifactual edge effects and providing smooth interaction contours amenable to direct interpretation [24]. Recently, several groups have used different QSAR models for prediction of binding affinity to the 5-HT_1A_ receptor of a series of structurally diverse compounds [13,25,26]. Because most of the QSAR models are limited in their applicability to compounds that have a common template structure, accurate prediction of binding affinity of novel 5-HT_1A_ receptors remains difficult. However, classification of binding affinity of diverse compounds to some extent may be feasible.

In this work, a new QSAR model for the binary classification of 153 5-HT_1A_ selective ligands has been developed with Adaboost-SVM. The variables were calculated by the E-Dragon 1.0 software, and the descriptors were pre-selected by particle swarm optimization (PSO) and stepwise multiple linear regression (Stepwise -MLR) methods.

## 2. Results and Discussion

### 2.1. Variable selection and model building

In the present work, the stepwise multiple linear regression (stepwise-MLR) and the particle swarm optimization (PSO) optimization techniques have been used for the selection of the most relevant descriptors from the pool of 77 topological descriptors depending on the compounds in the training set.

#### 2.1.1. Variable selection by stepwise-MLR

Stepwise-MLR is a popular technique that has been used on the training data set to select the most appropriate descriptors [27]. This method has been used for variable selection or model development in different systems [28–30]. We select significant descriptors using Stepwise-MLR procedure.

The pool of 119 topological descriptors has been calculated by using the software E-Dragon 1.0 for each compound in the training set. The pool of 42 topological descriptors with constant or near constant values inside each group is discarded. The eight most significant descriptors in the training set, which were selected by Stepwise-MLR are: MSD, MAXDN, MAXDP, PW4, PW5, PJI2, BAC, ICR, whose definitions are depicted in Table 1.

The eight variables compose of the variable subset of Stepwise-MLR-SVM and Stepwise-MLR-Adaboost-SVM model, respectively. The predicted result of training set and test set are listed in Table 2. The misclassified samples (marked by superscript ‘**’) of SVM and Adaboost-SVM are also listed. The same misclassified ones of SVM and Adaboost-SVM are a9, a10, e8, m8, n5 and n6. For the training set, when only SVM was used as the base classifier, the total accuracy was 0.991 (Table 3). When AdaBoost algorithm was used to boost the SVM classifier, after 100 iterations, the total accuracy was 0.991 (Table 3). For the testing set, for SVM only and the AdaBoost algorithm, the total accuracy was 0.775 and 0.825 (Table 3), respectively. Comparing with the only the SVM algorithm, it showed that better predicted accuracy was obtained by Stepwise-MLR-Adaboost-SVM. The predictive ability of Stepwise-MLR-Adaboost-SVM is raised 5.0%. The result implies that the AdaBoost algorithm can boost the SVM classifier, and let it make a stronger classifier.

#### 2.1.2. Variable selection by PSO

PSO is a population-based optimization tool. The system is initialized with a population of random solutions and searches for optima by updating generations. Unlike genetic algorithms (GA), PSO has no evolution operators, such as cross-over and mutation. Compared to GA, the advantages of PSO are that PSO is easy to implement and there are few parameters to adjust. In this paper, PSO has been used to find the optimized combinations of variables, from which one can extract the most related variables that capture maximally the information of the original variable blocks to establish the classification model.

The PSO algorithm maintains a population of 10 particles, *c*_1_=*c*_2_=1.8, *V_max_*=0.2, *X_max_*=1.0, *X_min_*=0.0 and *w* was linearly decreased from 1.0 to 0.2 during the 100 iterations. The variable selection methods have been used to select the most significant descriptors from the pool of 77 topological descriptors depending on the compounds in the training set. The selected descriptors by the method have been used to construct some models by using SVM and Adaboost-SVM techniques. These models can be shown as PSO-SVM and PSO-Adaboost-SVM. The selected variables are shown in Table 3 and their meaning in Table 2.

The seven variables compose of the variable subset of PSO-SVM and PSO-Adaboost-SVM model, respectively. The predicted result of training set and test set is listed in Table 1. The same misclassified ones of SVM and Adaboost-SVM are m8 and n4.

When PSO was used as the variable selection method and SVM was used as the base classifier, for training set, the total accuracy was 0.991 (Table 3); for testing, the total accuracy was 0.925 (Table 3).

When PSO was used as the variable selection method and AdaBoost algorithm was used to boost SVM classifier. In SVM, the kernel function was the Gaussian radial basis function (RBF) because of its good generalization and a few parameters, the RBF is defined as below: exp(−*γ*(*x* − *x_i_*)^2^), where γ is width of RDF. Optimized parameters of the SVM are: width of RBF γ=0.0001 and capacity parameter c=5000. The best total accuracy of training and test set was 1.000 and 0.950, respectively (Table 3). The total accuracy of training and test set was raised 0.9% and 2.5%, respectively.

Comparing the results of two different variable selection methods, PSO is a more suitable tool to optimize result of classification problem compared to SVM and AdaBoost-SVM algorithm.

### 2.2. Comparison with different models

The selection of this model as the best one obtained with the topological descriptors is because of the best global classification for training set and test set of the models obtained with this family of descriptors. The classification results for the training set and test set are illustrated in Table 3.

As can be seen from the table, the values of the predicted accuracy of high 5-HT_1A_ affinity compounds, the predicted accuracy of low 5-HT_1A_ affinity compounds and the total accuracy of the training and test set are higher than 0.82, 0.66 and 0.77, respectively. Therefore all the models used in comparison present a good predicted result. As can be seen from Table 3, the accuracy is 0.821 and 0.893 on the high 5-HT_1A_ affinity group for Stepwise-MLR -SVM and Stepwise-MLR -AdaBoost-SVM respectively, and 0.964, 0.964 for PSO-SVM and PSO-AdaBoost-SVM respectively, and on the low 5-HT_1A_ affinity group, the overall accuracy is 0.667, 0.667 for Stepwise-MLR -SVM, Stepwise-MLR -AdaBoost-SVM and 0.833, 0.917 for PSO-SVM, PSO-AdaBoost-SVM respectively. The total accuracy of training set for Stepwise-MLR-SVM, Stepwise-MLR-AdaBoost-SVM, PSO-SVM, PSO-AdaBoost-SVM is 0.991, 0.991, 0.991 and 1.000 respectively. The total accuracy of the test set for PSO-AdaBoost-SVM is 0.950, higher than that of Stepwise-MLR-SVM (0.775), Stepwise-MLR-AdaBoost-SVM (0.825) and PSO –SVM (0.925), respectively.

From the comparison of the four methods, it can be seen that performance of PSO-AdaBoost-SVM is better than that of Stepwise-MLR-SVM, Stepwise-MLR-AdaBoost-SVM and PSO-SVM, which implies that, extracting the most related variables that capture maximally the information of the original variable blocks is very important to raise predicted accuracy of the test set, and AdaBoost algorithm was used to boost SVM classifier. In Table 3, we have observed an interesting phenomenon: The predicted accuracy of the low 5-HT_1A_ affinity group is lower than that of the high 5-HT_1A_ affinity group. The reasons for this phenomenon are not clear, so perhaps some of compounds misclassified by every method need further experimental testing.

In the PSO-AdaBoost-SVM model, the percentages of false negatives and false positives in the test set are 3.6% (1/28) and 8.3% (1/12), respectively. False positives are those compounds without binding affinities of 5-HT_1A_ that are classified as active, and the false negatives are those compounds with binding affinities of 5-HT_1A_ that are classified as inactive (see Table 3). From a practical point of view, in the development of the classification model, it is considered more important to avoid false negatives compounds because those compounds will be rejected for their wrongly predicted property and therefore they will never be evaluated experimentally, and their true binding affinities of 5-HT_1A_ would never be discovered. On the contrary, the false positives compounds eventually will be detected.

### 2.3. Variable’s interpretation of the best model

The molecular structure can be represented using many theoretical descriptors from the literature, but we usually face the problem of selecting those which are the most representatives for the property under consideration. Topological indices have been widely used in the correlation of physicochemical properties of organic compounds. And it is also known as graph theoretical indices are descriptors that characterize molecular graphs and contain a large amount of information about the molecule, including the numbers of hydrogen and non-hydrogen atoms bonded to each non-hydrogen atom, the details of the electronic structure of each atom, and the molecular structural features.

From the best PSO-selected model, clearly the seven descriptors appearing in this model are the first Mohar index (TI1), distance/detour index (D/D), molecular electrotopological variation (DELC), 1-path Kier alpha-modified shape index (S1K), eccentricity (ECC), mean distance degree deviation (MDDD) and Balaban centric index (BAC).

Molecular branching and molecular cyclic structure are the two most visible structural elements that widely vary among molecules. Randić [31] constructed a new matrix D/DD, which has a finer discriminating power, particularly when one is interested in local molecular features, such as atomic, bond, or ring descriptors. At the same time, the D/DD matrix is more sensitive to the immediate and global environment of vertices or larger graph fragments. Hence, the distance/detour index (D/D) descriptor can discriminate 5-HT_1A_ selective ligands primely.

Balaban centric index (BAC) [32,33], is that the sum of the BF vector gives the centric index $\text{CI}=\sum_{i=1}^{n}BF_{i}$, where n denotes the number of vertices; *BF* is balance function, which is determined as follows $BF_{i}=\sum_{j=1}^{n}I_{j}V_{j}D_{ij}$, where *I_j_* is the intrinsic state of atoms *j*, *V_j_* is the vertex degree of atom *j*, and *D_ij_* is the distance between atoms *i* and *j*. The BF method is based on both topological and electronic information, which can be applied in molecules with polycyclic, multiple bond and heteroatom. When molecular skeletons are differently organized with respect to the graph center, molecular centricity becomes of importance [34]. Graph center and related parameters are useful for coding of molecular structure, as well as modeling QSAR. So, 5-HT_1A_ selective ligands can be distinguished primely using the BAC descriptor.

Molecular electrotopological variation (DELS) [35] is simply the sum over all atoms of the intrinsic state differences and could be a measure of total charge transfer in the molecule. The DELS is defined as follows $DELS=\sum_{i}\Delta I_{i}$, Δ*I_i_* is the field effect on the *i*th atom due to the perturbation of all other atoms as defined by Kier and Hall [36]: $\Delta I_{j}=\sum_{j}\frac{I_{i}-I_{j}}{{\left(d_{ij}+1\right)}^{2}}$, where, *I* is the atomic intrinsic state, *d* denotes the topological distance between the two considered atoms. The intrinsic state of an atom is calculated as the ratio between Kier-Hall atomic electronegativity and the vertex degree, i.e. the number of bonds of the atom, encoding information related to both partial charges of atoms and their topological position relative to the whole molecule. Mean distance degree deviation (MDDD) [37] is defined as follows $\Delta D(G)=\frac{1}{p}\sum_{v\in V(G)}\left|d(v)-\frac{2W(G)}{p}\right|$, where *G* is a finite connected graph without loops and multiple edges; *V*(*G*) is the set of vertices of graph *G* with cardinality *p* = |*V* (*G*)|; $W(G)=\frac{1}{2}\sum_{u,v\in V(G)}d(u,v)$ is the Wiener index of the graph G, where the distance *d*(*u*,*v*) between vertices *u,v* in graph *G* is the length of a simple path which joins the vertices *u* and *v* in the graph *G* and contains the minimal number of edges; $d(v)=\sum_{i=1}^{p}d(i,v)$ is called the atom eccentricity (ECC) of the atom *i*. In addition, S1K [38] is the Kier–Hall α-modified shape index which is a measure of the relative cyclicity of a compound. A decrease in the value indicates an increase in cyclicity with multi-cyclic compounds having lower values than monocyclic ones. TI1 is the first Mohar index, which is also important topological index. And it has been successfully applied to construct the QSAR model [39].

Regarding above reasons, the seven selected topological descriptors can identify primely different structure information of 5-HT_1A_ selective ligands. Hence, our PSO-Adaboost-SVM model relating to the topological descriptor predictor should be useful for classification of prediction inhibitory activities of the new synthetic 5-HT_1A_ selective ligands derivatives.

### 2.4. Comparison with other approaches

As we have previously explained, one of the objectives of the current work was to compare the reliability and applicability of the topological descriptors to describe the property under study as compared with other different descriptors. Consequently, we have developed other twelve models using the same data set that was included in the topological descriptor for the PSO-AdaBoost-SVM model. The results obtained with Constitutional, Information indices, Randic molecular profiles, RDF, WHIM, Topological, 2D autocorrelation Indices, Burden eigenvalues, Eigenualue based indices, Geometrical, 3D-MoRSE, and GETAWAY descriptors [40], are given in Table 4. These descriptors have been calculated by the software E-Dragon 1.0. The comparisons have been done based on classification results, and the predictive capability of the generated models.

As can be seen from Table 4, the value of TA of test set is lower than 90.1% for all approaches except the topological descriptor which has a TA equal to 95.0%. This approach also yields the best value for TA of the training set and percentages of false positives in the test set which has the lowest values in comparison with the rest of the approaches. Additionally, the topological descriptor presents better the percentages of false negatives in the test set, except for information indices descriptor, but information indices descriptor has worse predictive capability for low 5-HT_1A_ affinity compounds (only 50.0%). In this sense, other families of descriptors such as Constitutional, RDF, Topological, 2D autocorrelation Indices, Geometrical, 3D-MoRSE, GETAWAY, Randic molecular profiles, WHIM, Burden eigenvalues, and Eigenualue based indices have presented similar percent of high 5-HT_1A_ affinity compounds classification in the test set (92.9, 89.3, 96.4, 92.9, 89.3, 96.4, 85.7, 96.4, 92.9, 89.3, and 92.9%, respectively), while they have shown worse classification for low 5-HT_1A_ affinity compounds in the test set. So, the topological descriptor for the PSO-AdaBoost-SVM model, not only overtakes the others models in the predictive accuracy of false negatives, but the total accuracy of compounds in the test set is the best. For all these reasons, we have considered that the PSO-AdaBoost-SVM method with topological descriptor can be a useful tool for classification of 5-HT_1A_ selective ligands on the basis of their binding affinities.

## 3. Experimental and the Theory of the Modeling Methods

### 3.1. Experimental

#### 3.1.1. Data sets

**Figure 1.**
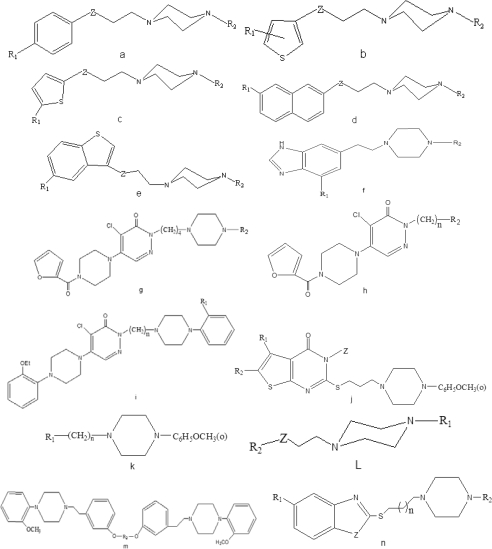
Generic structures for the 153 compounds used in this study.

The studied compounds are 153 5-HT_1A_ selective ligands, which were taken from the literature [1,11–16] and their generic structures are shown in Figure 1. The inhibition constant (*K*_i_) is obtained from the IC_50_ value by the Cheng-Prusoff equation [39]. The tested compounds are selective 5-HT_1A_ ligands showing *K*_i_ values from 0.094 to 5000 nM. For analysis purposes, p*K_i_* values are used as the dependent variables and are given in Table 1. The compounds studied in our investigation are more diverse. It is difficult to build a QSAR model by their activity values because there is a very low similarity of the complex structure. So, the compounds are divided into two classifications according to 5-HT_1A_ selective ligands binding affinities: high 5-HT_1A_ affinity and low 5-HT_1A_ affinity. Compounds with p*K_i_* values>6.7 are assumed as high 5-HT_1A_ affinity compounds and p*K_i_* values≤6.7 are assumed as low 5-HT_1A_ affinity compounds [13], which are represented by ‘1’, and ‘−1’, respectively. The whole data set with 153 compounds is randomly divided into training set and test set. The training set is used to adjust the parameters of the models. The test set is used to evaluate the performance of the models once they are built. The training set consists of 113 compounds (including 73 high 5-HT_1A_ affinity compounds and 40 low 5-HT_1A_ affinity compounds), and the test set contains 40 compounds (including 28 high 5-HT_1A_ affinity compounds and 12 low 5-HT_1A_ affinity compounds).

#### 3.1.2. Descriptor calculation

To develop a QSAR model, molecular structures need to be represented using molecular descriptors, which encode structural information. The calculation process of the descriptors involves the following steps: the structures of the compounds are drawn using Molinspiration WebME Editor [42] and saved as a.smi files. Then the a.smi files are transferred into the software E-Dragon 1.0 [43] to calculate zero, one, two and three dimensional structural descriptors. The software E-Dragon 1.0 can calculate Constitutional, Information indices, Randic molecular profiles, RDF, WHIM, Topological, 2D autocorrelation Indices, Burden eigenvalues, Eigenualue based indices, Geometrical, 3D-MoRSE, and GETAWAY descriptors. And the descriptors have been successfully used in various QSAR/QSPR researches [44–46]. In the pre-reduction step, the calculated descriptors are searched for constant values for all molecules and those detected descriptors are removed, and the others calculated descriptors would be used as original variable set.

### 3.2. The theory of the modeling methods

#### 3.2.1. Theory of PSO

Particle swarm optimization (PSO) is an optimization algorithm, which simulates the movement and flocking of birds [47]. Similar to other population-based algorithms, such as evolutionary algorithms, PSO can solve a variety of difficult optimization problems but has shown a faster convergence rate than other evolutionary algorithms on some problems [48]. The other advantage of PSO is that it has very few parameters to adjust, which makes it particularly easy to implement.

PSO is based on the fact that in order to reach the optimum solution in a multidimensional space, a population of particles is created whose present coordinate determines the cost function to be minimized. After each iteration the new velocity and hence the new position of each particle is updated on the basis of a summated influence of each particle’s present velocity, distance of the particle from its own best performance, achieves so far during the search process and the distance of the particle from the leading particle, i.e. the particle which at present is globally the best particle producing till now the best performance, i.e. minimum of the cost function achieved so far.

Let *x* and *v* denote a particle position and its corresponding velocity in a search space, respectively. Therefore, the *i* th particle in the d-dimensional search space can be represented as *x_i_* = (*x_i1_*, *x_i2_*,…, *x_id_*) and *v_i_* = (*v_i1_*, *v_i2_*,…,*v_id_*), respectively. Each particle has its own best position (*pbest*), *pb_i_*= (*pb_i1_*, *pb_i2_*, …, *pb_id_*) corresponding to the personal best objective value obtained so far at time t. The index of the best particle among all the particles in the group is represented by the *pb_g_*, which represents the best particle found so far at time t. The new velocity of each particle is calculated as follows:

(1) $$v_{ij}(t+1)=w\times v_{ij}(t)+c_{1}r_{1}(pb_{ij}-x_{ij}(t))+c_{2}r_{2}(pb_{gj}-x_{ij}(t)),i=1,2,\cdots m,j=1,2,\cdots d.$$

where, *m* is the number of particles in a group; *d* is the number of members in a particle; *c*_1_, *c*_2_ are constants, which control how far a particle will move in a single iteration; *r*_1_, *r*_2_ are two independent random numbers uniformly distributed in the range of [0, 1] and *w* is the inertia weight. A larger inertia weight facilitates global exploration and a smaller inertia weight tends to facilitate local exploration to fine-tune the current search area [49,50].

Thus, the position of each particle is updated iteration according to the following equation:

(2) $$x_{ij}(t+1)=x_{ij}(t)+v_{ij}(t+1),i=1,2,\cdots m,j=1,2,\cdots d.$$

where *v_i_* (t) is the velocity of a particle *i* at iteration t, $v_{j}^{\text{min}}\leq v_{ij}(t)\leq v_{j}^{\text{max}}$ and *x_i_* (t) is the current position of a particle *i* at iteration t. Generally, the value of each component in *v_i_* by Equation (1) can be clamped to the range [−*v*_max_, *v*_max_] to control excessive roaming of particles outside the search space. Then the particle flies toward a new position according to Equation (2). This process is repeated until a user-defined termination criterion is reached, and the termination criterion is determined according to whether the maximum iteration or a designated value of the fitness function.

#### 3.2.2. Theory of AdaBoost algorithm

Boosting is a learning process to make a strong classifier by combining multiple weak classifiers. The weak classifier just has slightly better performance than random classification [51]. And boosting demands prior knowledge of the accuracy of the weak learners. In 1997, Freund *et al.* proposed a method that does not have this requirement [52]. This method is called Adaptive boosting (AdaBoost). AdaBoost algorithm is the most frequently used Boosting method. Depending on the purpose and data structure, variants on AdaBoost algorithm have been developed, such as Discrete AdaBoost, Real AdaBoost and AdaBoost.MH [53]. Here the Discrete AdaBoost has been employed. The other two have been described in detail [48] and will not be repeated here.

Suppose there is a training data set with N samples to in two classes. The two classes are defined as *y*∈{−1,1}, 1 and −1 corresponding to high and low, respectively. A sequence of N training examples (labelled instances) (*x*_1_, *y*_1_), ..., (*x_N_*, *y_N_*) is drawn randomly from *X*×*Y* according to distribution ζ. We use boosting to find a hypothesis *h_f_* which is consistent with most of the sample (i.e., *h_f_* (*x_i_*)=*y_i_* for most 1≤*i*≤*N*).

The Discrete AdaBoost algorithm can be implemented as follows [51–54]:

**Step 1** Distribution D over the *N* training examples, initializes the weight vector:
  (3) $$w_{i}^{1}=\frac{1}{N}\;\text{for}\;i=1,2,...,N$$
**Step 2** Do for *t* = 1, 2,…, *T*
  (4) $$\text{Step}\;2\text{a}\;\text{Set}\;p^{t}=\frac{w^{t}}{\sum_{i=1}^{N}w_{i}^{t}}$$

Select a data set with *N* samples from the original training set. The chance for a sample to be selected is related to the distribution of the weights *p^t^*. A sample with a higher weight has a higher probability to be selected.

Step 2b Call Weaklearn *F_t_*(*x*), which is done with SVM in our case, with the training set base on the current distribution *p^t^* and get back a hypothesis *h_t_*(*x*): *F_t_*(*x*)→*h_t_*(*x*).

Step 2c Calculate the sum of the weighted errors of all training samples according to hypothesis *h_t_*(*x*).

(5) $$\varepsilon_{t}=\frac{\sum_{i=1}^{N}p_{i}^{t}\left|h_{t}(x_{i})-y_{i}\right|}{2}$$

Step 2d Update weights of the correctly classified samples and let the misclassified samples unchanged among all the original training samples.

(6) $$w_{i}^{t+1}=w_{i}^{t}\beta_{t},i\;\text{refers to the samples that are correctly classified}.$$

(7) $$\text{Where}\;\beta_{t}=\frac{1}{2}\text{ln}\frac{\varepsilon_{t}}{1-\varepsilon_{t}}$$

According to formula (6) and (7) the weights of the samples that are correctly classified are decreased while the weights of the misclassified samples are unchanged.

Step 2e The confidence index of hypothesis *h_t_*(*x*) is calculated as:

(8) $$\alpha_{t}=\text{log}\frac{1}{\beta_{t}}$$

The lower the weighted error made by hypothesis *h_t_*(*x*) on the training samples, the higher the confidence index of the hypothesis *h_t_*(*x*).

Step 2f If ɛ_t_<0.5 or *t* ≤ *T*, repeat step (1) ~ step(5); otherwise, stop and *T* = *t* − 1.

After *T* iterations in **Step 2**, there is *T* hypothesis *h_t_*(*x*)s which is associated with *T* base learning algorithm *F_t_*(*x*)s.

**Step 3** The performance of Discrete AdaBoost is evaluated by a test set. For a sample *j* of the test set, the final prediction is the combined prediction obtained from the *T* learners. Each prediction is multiplied by the confidence index of the corresponding learner *h_t_* (*x*). The higher confidence index of a learner *h_t_* (*x*), the higher its role in the final decision:

(9) $$y_{j}=\text{sign}\;\left(\sum_{t=1}^{T}\alpha_{t}h_{t}(x_{j})\right)$$

where $sign\;(x_{i})=\left\{ \begin{array}{l} 1,\;if\;x_{i}\geq 0\\ -1,\;if\;x_{i}<0 \end{array}\right.$

#### 3.2.3. Methodology

After the descriptors are selected using two variable selection methods (Stepwise-MLR and PSO), the next step is to build the classification model using SVM (support vector machines) [55] and Adaboost-SVM methods, respectively. These models can be shown as Stepwise-MLR-SVM, Stepwise-MLR-Adaboost-SVM, PSO-SVM and PSO-Adaboost-SVM. As the Adaboost algorithm has been depicted in the previous section, we only give a simple description on the theory of SVM.

The support vector machine (SVM), was introduced by Vapnik [56] as a novel type of learning machine, gaining popularity due to many attractive features and promising empirical performance. Originally, SVM was developed for pattern recognition problems. And now, with the introduction of a *ɛ*-insensitive loss function, SVM has been extended to solve nonlinear regression estimation and time-series prediction and excellent performances have been obtained [57].

For the classification problem, in brief, this involves the optimization of Lagrangian multipliers α_i_ with constraints 0 ≤α_i_≤ C and Σα_i_*y_i_*=0 to yield a decision function:

(10) $$f(x)=\text{sign}\;\left(\sum_{i=1}^{l}y_{i}\alpha_{i}K(x,x_{i})+b\right)$$

Where sign (*u*) implies a sign function which returns +1 when *u*>0, and −1 when *u*≤0; *y_i_* is input class labels that take a value of −1 or +1, *x_i_* is a set of descriptors; and *K*(x, x*_i_*) is a kernel function, whose value is equal to the inner product of two vectors x and x*_i_* in the feature space Φ(x) and Φ(x*_i_*). That is, *K*(x, x*_i_*)= Φ(x). Φ(x*_i_*). Any function that satisfies Mercer’s condition can be used as the kernel function.

In SVM, we chose c-SVC [55] as the base classifier and the kernel function was the Gaussian radial basis function (RBF) function.

All the algorithms were written in MATLAB and run on a personal computer (Intel(R) Pentium(R) 4 / 3.20 GHz, 1.00GB RAM).

#### 3.2.4. The evaluation of prediction power

In this study, the quality of a model is assessed by several statistical measures, including false-negative (FN), false-negative rate (FNR), false-positive (FP), false-positive rate (FPR), and total accuracy (TA).

**FP:** false-positives, the number of chemicals predicted to be active but inactive in the assay,

**FN:** false-negatives, the number of chemicals predicted to be inactive but active in the assay, FPR and FNR defined as follows:

(11) $$\text{FPR}=\frac{\text{FP}}{N_{-}}$$

(12) $$\text{FNR}=\frac{\text{FN}}{N_{+}}$$

(13) $$\text{TA}=\frac{\text{N}-\text{FP}-\text{FN}}{N}$$

where, *N*_−_ denotes the total number of inactive chemicals in the data set, *N*_+_ denotes the total number of active chemicals in the data set, *N* denotes the total number of active and inactive chemicals in the data set.

## 4. Conclusions

In this work, AdaBoost-SVM has been developed for the QSAR analysis of 5-HT_1A_ selective ligands. The binding affinities of 153 5-HT_1A_ selective ligands were classified. The variables for the molecular descriptors were determined by PSO. Compared with other descriptors in the Adaboost-SVM model and SVM model, the Topological descriptor composed of AdaBoost-SVM model exhibited the best prediction accuracy (the accuracy for the overall data set reached 95.0%). The combination of Adaboost-SVM and PSO gives a useful tool for QSPR/QSAR studies and classification investigations.

## Figures and Tables

**Table 1.** Symbols of topological descriptors for molecular descriptors used in different models and their definitions.

**Symbols**	**Descriptor definition**
MSD	mean square distance index (Balaban)
MAXDN	maximal electrotopological negative variation
MAXDP	maximal electrotopological positive variation
PW4	path/walk 4 - Randic shape index
PW5	path/walk 5 - Randic shape index
PJI2	2D Petitjean shape index
BAC	Balaban centric index
ICR	radial centric information index
TI1	first Mohar index TI1
D/D	distance/detour index
DELS	molecular electrotopological variation
S1K	1-path Kier alpha-modified shape index
ECC	eccentricity
MDDD	mean distance degree deviation

**Table 2.** Compounds’ structures, p*K*_i_ values and their corresponding classifications.

No.	R_1_	R_2_	Z	n	p*K*_i_	Class				
						Exp.	MS	MAS	PS	PAS
a1	H	2-Methoxyphenyl	CHOH	–	7.32	1	1	1	1	1
a2	H	2-Methoxyphenyl	CHO-4-CF_3_C_6_H_4_	–	6.35	−1	−1	−1	−1	−1
a3*	H	2-Methoxyphenyl	CNOH	–	7.76	1	−1**	1	1	1
a4	H	4-Chlorophenyl	CO	–	6.10	−1	−1	−1	−1	−1
a5*	H	4-Chlorophenyl	CHO-4-CH_3_C_6_H_4_	–	5.84	−1	−1	−1	−1	−1
a6	H	4-Chlorophenyl	CHO-3,4-OCH_2_OC_6_H_3_	–	6.26	−1	−1	−1	−1	−1
a7	H	4-Methoxyphenyl	CO	–	5.30	−1	−1	−1	−1	−1
a8	H	4-Methoxyphenyl	CHOH	–	5.30	−1	−1		−1	−1
a9*	H	2-Chlorophenyl	CO	–	6.74	1	−1**	−1**	1	1
a10*	H	2-Chlorophenyl	CHOH	–	6.94	1	−1**	−1**	1	1
a11	H	2-Chlorophenyl	CHO-4-CF_3_C_6_H_4_	–	5.30	−1	−1	−1	−1	−1
a12	H	4-Fluorophenyl	CO	–	6.10	−1	−1	−1	−1	−1
a13	H	4-Fluorophenyl	CHO-4-CF_3_C_6_H_4_	–	5.30	−1	−1	−1	−1	−1
a14	H	2-Pyridyl	CO	–	7.30	1	1	1	1	1
a15*	H	2-Pyridyl	CHOH	–	6.81	1	1	1	1	1
a16	H	4-Nitrophenyl	CO	–	5.30	−1	−1	−1	−1	−1
a17	H	4-Nitrophenyl	CHOH	–	5.30	−1	−1	−1	−1	−1
a18	H	4-Nitrophenyl	CHO-4-CF_3_C_6_H_4_	–	5.30	−1	−1	−1	−1	−1
a19	Phenyl	2-Methoxyphenyl	CO	–	5.44	−1	−1	−1	−1	−1
a20*	Phenyl	2-Methoxyphenyl	CHO-4-CF_3_C_6_H_4_	–	5.30	−1	−1	−1	1**	−1
a21	Methoxy	2-Methoxyphenyl	CO	–	5.76	−1	−1	−1	−1	−1
a22	Methoxy	2-Methoxyphenyl	CHOH	–	6.49	−1	−1	−1	−1	−1
a23	Methoxy	2-Methoxyphenyl	CHO-4-CF_3_C_6_H_4_	–	6.00	−1	−1	−1	−1	−1
a24	H	2-Methoxyphenyl	CO	–	7.30	1	1	1	1	1
a25	H	4-Chlorophenyl	CHOH	–	6.10	−1	−1	−1	−1	−1
a26	H	4-Methoxyphenyl	CHO-4-CF_3_C_6_H_4_	–	5.30	−1	−1	−1	−1	−1
a27	H	2-Pyrimidyl	CO	–	6.92	1	1	1	1	1
a28	H	2-Pyrimidyl	CHO-4-CF_3_C_6_H_4_	–	5.80	−1	−1	−1	−1	−1
a29	H	2-Pyridyl	CHO-4-CF_3_C_6_H_4_	–	5.80	−1	−1	−1	−1	−1
a30	Phenyl	2-Methoxyphenyl	CHOH	–	6.07	−1	−1	−1	−1	−1
b1	H	2-Methoxyphenyl	CHOH	–	7.30	1	1	1	1	1
b2*	H	2-Methoxyphenyl	CHO-4-CF_3_C_6_H_4_	–	6.59	−1	−1	−1	−1	−1
b3	H	2-Methoxyphenyl	CNOH	–	8.19	1	1	1	1	1
b4*	H	4-Chlorophenyl	CO	–	6.15	−1	−1	−1	−1	−1
b5	H	2-Chlorophenyl	CO	–	6.70	−1	−1	−1	−1	−1
b6	H	2-Chlorophenyl	CHOH	–	6.70	−1	−1	−1	−1	−1
b7	H	1-Naphthyl	CO	–	7.46	1	1	1	1	1
b8*	2,5-Dimethyl	2-Methoxyphenyl	CO	–	8.30	1	1	1	1	1
b9*	2,5-Dimethyl	2-Hydroxyphenyl	CO	–	8.12	1	−1**	1	1	1
b10	2,5-Dimethyl	2-Hydroxyphenyl	CHOH	–	7.05	1	1	1	1	1
b11	2,5-Dimethyl	1-Naphthyl	CO	–	7.00	1	1	1	1	1
b12*	H	H	CO	–	7.80	1	1	1	1	1
b13	H	H	CHOH	–	5.56	−1	−1	−1	−1	−1
b14	2,5-Dimethyl	2,5-Dimethyl	CHOH	–	7.92	1	1	1	1	1
c1	H	2-Methoxyphenyl	CO	–	8.00	1	1	1	1	1
c2	H	2-Methoxyphenyl	CHOH	–	7.72	1	1	1	1	1
c3	H	4-Chlorophenyl	CO	–	5.30	−1	−1	−1	−1	−1
c4	H	4-Chlorophenyl	CHOH	–	5.30	−1	−1	−1	−1	−1
c5	5-Methyl	2-Methoxyphenyl	CO	–	7.76	1	1	1	1	1
c6	5-Methyl	2-Methoxyphenyl	CHOH	–	7.47	1	1	1	1	1
c7	5-Nitro	2-Methoxyphenyl	CO	–	6.47	−1	−1	−1	−1	−1
d1	H	2-Methoxyphenyl	CHOH	–	6.38	−1	−1	−1	−1	−1
d2	H	4-Chlorophenyl	CO	–	5.30	−1	−1	−1	−1	−1
d3*	H	4-Chlorophenyl	CHOH	–	5.30	−1	−1	−1	−1	−1
d4*	H	2-Methoxyphenyl	CO	–	6.60	−1	−1	−1	−1	−1
e1	H	2-Methoxyphenyl	CO	–	7.36	1	1	1	1	1
e2	H	2-Methoxyphenyl	CHOH	–	7.70	1	1	1	1	1
e3	H	4-Chlorophenyl	CO	–	5.30	−1	−1	−1	−1	−1
e4	H	4-Chlorophenyl	CHOH	–	5.30	−1	−1	−1	−1	−1
e5*	H	2-Hydroxyphenyl	CO	–	6.96	1	−1**	1	1	1
e6	H	2-Hydroxyphenyl	CHOH	–	7.74	1	1	1	1	1
e7	H	4-Chloro-2-methoxyphenyl	CO	–	6.30	−1	−1	−1	−1	−1
e8*	H	4-Chloro-2-methoxyphenyl	CHOH	–	6.44	−1	1**	1**	−1	−1
e9	H	4-Fluoro-2-methoxyphenyl	CHOH	–	6.30	−1	−1	−1	−1	−1
e10*	H	1-Naphthyl	CO	–	7.00	1	1	1	1	1
e11*	H	4-Fluoro-2-methoxyphenyl	CO	–	6.30	−1	−1	−1	−1	−1
f1	Cl	Phe	–	–	7.05	1	−1**	1	1	1
f2	Cl	2-MeOPhe	–	–	8.28	1	1	1	1	1
f3	Cl	2-ClPhe	–	–	7.34	1	1	1	1	1
f4	Cl	2-CF_3_Phe	–	–	7.68	1	1	1	1	1
f5	Cl	Pyrimidin-2-yl	–	–	6.28	−1	−1	1**	−1	−1
f6	Br	Phe	–	–	7.62	1	1	1	1	1
f7	Br	2-MeOPhe	–	–	8.85	1	1	1	1	1
f8*	Br	2-ClPhe	–	–	9.85	1	1	1	1	1
f9	Br	2-CF_3_Phe	–	–	7.99	1	1	1	1	1
f10	Br	Pyrimidin-2-yl	–	–	6.89	1	1	1	1	1
f11	–	Phe	–	–	6.71	1	1	1	1	1
f12	–	2-MeOPhe	–	–	7.69	1	1	1	1	1
f13	–	2-ClPhe	–	–	6.61	−1	−1	−1	1**	−1
f14	–	2-CF_3_Phe	–	–	7.48	1	1	1	1	1
g1*	–	2-isopropoxyphenyl	–	–	8.89	1	1	1	1	1
g1*	–	2-ethoxyphenyl	–	–	7.96	1	1	1	1	1
g1	–	2,3-dihydrobenzo [1,4]dioxin-2-yl-methyl	–	–	6.63	−1	−1	−1	−1	−1
g1	–	2-pyrimidyl	–	–	6.68	−1	−1	−1	−1	−1
g1	–	3-chlorophenyl	–	–	7.77	1	1	1	1	1
g1	–	3-trifluoromethylphenyl	–	–	6.86	1	1	1	1	1
h1	–	4-(3-chlorophenyl)piperazin-1-yl	–	3	6.21	−1	−1	−1	−1	−1
h2	–	4-(3-trifluorophenyl)piperazin-1-yl	–	3	6.93	1	1	1	1	1
h3	–	4-pyridin-2-yl-piperazin-1-yl	–	3	5.94	−1	−1	−1	−1	−1
h4	–	4-(2-isopropoxyphenyl)piperazin-1-yl	–	2	8.70	1	1	1	1	1
i1*	OEt	–	–	4	8.30	1	1	1	1	1
i2	OEt	–	–	7	8.49	1	1	1	1	1
i3	O*i*Pr	–	–	4	8.41	1	1	1	1	1
i4	O*i*Pr	–	–	7	8.14	1	1	1	1	1
j1*	Me	Me	NH_2_	–	9.80	1	1	1	1	1
j2	Me	COOC_2_H_5_	NH_2_	–	8.87	1	1	1	1	1
j3	Me	C_2_H_5_	NH_2_	–	9.72	1	1	1	1	1
j4*	H	Me	C_2_H_5_	–	8.48	1	1	1	1	1
j5	Me	Me	C_2_H_5_CH= CH_2_	–	7.90	1	1	1	1	1
j6	Me	Me	NHCOCH_3_	–	8.36	1	1	1	1	1
j7*	Me	Me	H	–	7.88	1	1	1	1	1
j8	Me	Me	Me	–	8.79	1	1	1	1	1
j9*	Me	Me	NHC_6_H_5_	–	6.57	−1	−1	−1	−1	−1
k1	–	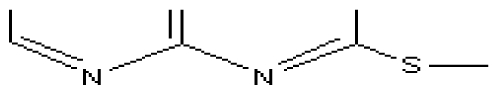	–	3	9.42	1	1	1	1	1
k2*	–	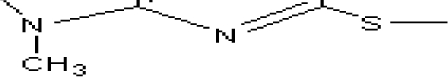	–	3	9.09	1	1	1	1	1
k3	–	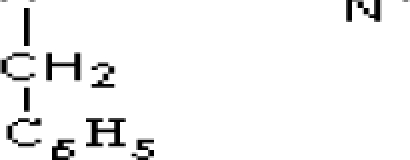	–	3	8.62	1	1	1	1	1
k4*	–	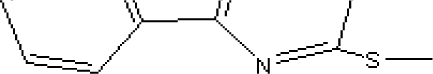	–	3	8.43	1	1	−1**	1	1
k5	–	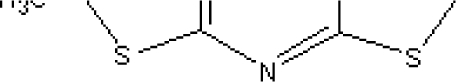	–	1	5.82	−1	−1	−1	−1	−1
k6	–	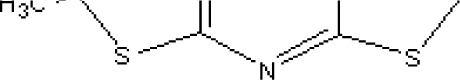	–	3	8.43	1	1	1	1	1
k7	–	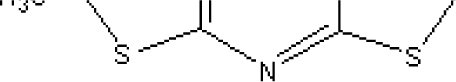	–	2	7.64	1	1	1	1	1
k8	–	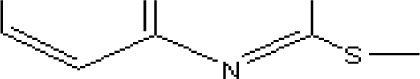	–	3	9.57	1	1	1	1	1
L1*	2-OCH_3_-phenyl	4-CH_3_-phenyl	C=O	–	7.00	1	1	1	1	1
L2	2-OCH_3_-phenyl	4-CH_3_-phenyl	CHOH	–	7.40	1	1	1	1	1
L3	2-OCH_3_-phenyl	2-CH_3_-phenyl	C=O	–	7.60	1	1	1	1	1
L4*	2-OCH_3_-phenyl	2-CH_3_-phenyl	CHOH	–	7.67	1	1	1	1	1
L5	2-OCH_3_-phenyl	2,4-di-CH_3_-phenyl	C=O	–	7.38	1	1	1	1	1
L6*	2-OCH_3_-phenyl	2,4-di-CH_3_-phenyl	CHOH	–	7.05	1	1	1	1	1
L7	2-OH-phenyl	2,4-di-CH_3_-phenyl	C=O	–	6.94	1	1	1	1	1
L8	2-OH-phenyl	2,4-di-CH_3_-phenyl	CHOH	–	6.96	1	1	1	1	1
L9	1-Naphtyl	2,4-di-CH_3_-phenyl	C=O	–	6.30	−1	−1	−1	−1	−1
m1	–	(CH_2_)_2_	–	–	8.66	1	1	1	1	1
m2	–	(CH_2_)_3_	–	–	8.25	1	1	1	1	1
m3	–	(CH_2_)_4_	–	–	9.05	1	1	1	1	1
m4*	–	(CH_2_)_5_	–	–	8.76	1	1	1	1	1
m5	–	(CH_2_)_6_	–	–	8.80	1	1	1	1	1
m6	–	(CH_2_)_8_	–	–	8.32	1	1	1	1	1
m7*	–	(CH_2_)_10_	–	–	7.55	1	1	1	1	1
m8*	–	(CH_2_)1_2_	–	–	6.60	−1	1**	1**	1**	1**
m9*	–	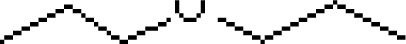	–	–	9.21	1	1	1	1	1
m10	–	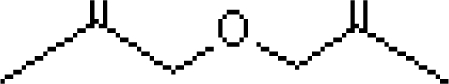	–	–	8.56	1	1	1	1	1
n1	H	2-CH_3_OC_6_H_4_	NH	0	7.48	1	1	1	1	1
n2	H	2-CH_3_OC_6_H_4_	S	0	7.51	1	1	1	1	1
n3*	H	2-CH_3_OC_6_H_4_	O	0	7.17	1	1	1	1	1
n4*	H	2-NO_2_C_6_H_4_	NH	0	7.02	1	1	1	−1**	−1**
n5*	H	2-NO_2_C_6_H_4_	S	0	5.77	−1	1**	1**	−1	−1
n6*	H	2-NO_2_C_6_H_4_	O	0	6.49	−1	1**	1**	−1	−1
n7	H	2-CH_3_OC_6_H_4_	NH	1	9.00	1	1	1	1	1
n8	H	2-CH_3_OC_6_H_4_	S	1	9.54	1	1	1	1	1
n9	H	2-CH_3_OC_6_H_4_	O	1	9.26	1	1	1	1	1
n10	H	2-NO_2_C_6_H_4_	NH	1	7.78	1	1	1	1	1
n11	H	2-NO_2_C_6_H_4_	S	1	8.02	1	1	1	1	1
n12	H	2-NO_2_C_6_H_4_	O	1	7.85	1	1	1	1	1
n13	H	Pyridin-2-yl	S	1	9.11	1	1	1	1	1
n14*	H	Pyridin-2-yl	O	1	8.80	1	1	1	1	1
n15	H	Pyrimidin-2-yl	S	1	8.60	1	1	1	1	1
n16	H	Pyrimidin-2-yl	O	1	7.83	1	1	1	1	1
n17	H	2-CH_3_OC_6_H_4_	NCH_3_	1	9.57	1	1	1	1	1
n18	Cl	2-CH_3_OC_6_H_4_	S	1	9.00	1	1	1	1	1
n19	Cl	2-CH_3_OC_6_H_4_	O	1	9.06	1	1	1	1	1
n20*	H	2-CH_3_OC_6_H_4_	S	2	9.57	1	1	1	1	1
n21	H	2-CH_3_OC_6_H_4_	O	2	10.03	1	1	1	1	1
n22	H	2-CH_3_OC_6_H_4_	S	4	8.89	1	1	1	1	1
n23	H	2-CH_3_OC_6_H_4_	O	4	9.28	1	1	1	1	1

1, high 5-HT_1A_ affinity compounds; −1, low 5-HT_1A_ affinity compounds.

* Test set;

** Misclassified compounds. MS, MLR-SVM; MAS, MLR- AdaBoost-SVM; PS, PSO-SVM; PAS, PSO- AdaBoost-SVM.

**Table 3.** The results of training set and test set for the four algorithms.

**Algorithm**	**Variables**	**Training set**			**Test set**		
		1-FNR	1-FPR	T A	1-FNR	1-FPR	TA
MLR-SVM	MSD,MAXDN,MAXDP,PW4, PW5, PJI2,BAC,ICR	0.986	1.000	0.991	0.821	0.667	0.775
MLR-AdaBoost-SVM		1.000	97.5	0.991	0.893	0.667	0.825
PSO-SVM	TI1, D/D, DELS, S1K,ECC, MDDD, BAC	1.000	97.5	0.991	0.964	0.833	0.925
**PSO-AdaBoost-SVM**		**1.000**	**1.000**	**1.000**	**0.964**	**0.917**	**0.950**

FNR, false-negative rate; FPR, false- positive rate; TA, total accuracy.

**Table 4.** The results of different models for the PSO-AdaBoost-SVM algorithm.

**Model**	**Variables**	**Training set Total**			**Test set Total**		

		**%, 1**	**%, −1**	**Total Accuracy (%)**	**%, 1**	**%, −1**	**Total Accuracy (%)**
Constitutional	Se, Mv, ARR, nC	94.5	77.5	88.5	92.9	50.0	80.0
RDF	RDF010u, RDF045u, RDF115m, RDF070v, RDF090v, RDF065e, RDF150p	95.9	85.0	92.0	89.3	91.7	90.0
**Topological**	TI1, D/D, DELS, S1K, ECC, MDDD, BAC	**100.0**	**100.0**	**100.0**	**96.4**	**91.7**	**95.0**
2D	ATS8m, ATS1e, MATS4m, GATS4m, GATS1v, GATS4e, GATS5p	98.6	97.5	98.2	92.9	75.0	87.5
Geometrical	AGDD, SPAN	100.0	100.0	100.0	89.3	66.7	82.5
3D MoRSE	Mor02u, Mor06u, Mor22m, Mor25m, Mor05v, Mor16v, Mor32v, Mor02e, Mor26p	98.6	95.0	97.3	96.4	66.7	87.5
GETAWAY	HTu, H6m, HATS3m, HATS5m, R5u, RTv, R7v+	100.0	100.0	100.0	85.7	83.3	85.0
Information indices	IDET, TIC0, SIC0, TIC5	98.6	100.0	99.1	100.0	50.0	85.0
Randic molecular profiles	DP19, SP01, SP09, SP18	97.3	75.0	89.4	96.4	66.7	87.5
WHIM	L1u, L2v, G3p, Te, Ts	100.0	100.0	100.0	92.9	58.3	82.5
Burden eigenvalues	BELm5, BEHv3, BELv1, BELv3, BEHe3	89.0	72.5	83.2	89.3	66.7	82.5
Eigenuaulue based indices	Eig1p, SEigZ, AEige, VEZ2, VRZ1, VRp2	100.0	100.0	100.0	92.9	75.0	87.5

1, high 5-HT_1A_ affinity compounds; −1, low 5-HT_1A_ affinity compounds.
